# Right patient to the right place: the impact of a 6-year regional trauma centre-led prehospital education programme on EMS triage and patient outcomes

**DOI:** 10.1186/s12873-025-01321-w

**Published:** 2025-08-20

**Authors:** Donghwan Choi, Yo Huh, Byung Hee Kang, Sora Kim, Seoyoung Song, Kyoungwon Jung, Hohyung Jung

**Affiliations:** 1 Division of Trauma Surgery, Department of Surgery, Ajou University School of Medicine, 164 Worldcup-ro, Yeongtong-gu, Suwon-si, Gyeonggi-do 16499 Republic of Korea; 2 Regional Trauma Center of Southern Gyeonggi Province, Ajou University School of Medicine, Suwon, Republic of Korea

**Keywords:** Undertriage, Prehospital triage, Performance improvement, Regional trauma system, EMS provider education

## Abstract

**Background:**

In regional trauma systems, emergency medical service (EMS) providers play a crucial role by performing prehospital triage for severely injured patients and transporting them to regional trauma centres. Since 2016, a regional trauma centre has provided prehospital medical guidance to EMS providers through a trauma hotline, facilitated by a trauma surgeon, to guide field triage, treatment, and transport. This study analysed the effects and clinical outcomes of a regional trauma centre-led performance improvement programme that followed closed-loop principles for EMS providers.

**Methods:**

We collected data from regional trauma centre databases (2016–2021) of patients with trauma with Injury Severity Scores > 15 in the Gyeonggi Province. The primary outcome was the undertriage rate, and the secondary outcomes were in-hospital mortality, hospital length of stay (LOS), intensive care unit LOS, and duration of mechanical ventilation. After severity and demographic adjustments through propensity score matching, clinical outcomes were analysed using the Mann–Whitney U and chi-squared test. The results were expressed as medians with interquartile ranges.

**Results:**

Among the 3017 patients included in the 6-year study period, correct triage and undertriage were performed in 2528 and 489, respectively. From 2016 to 2021, prehospital medical guidance and feedback provision increased from 432 times (32.1%) to 1505 times (96.8%) (*p* < 0.001); the undertriage rate decreased from 32.7% (*n* = 55/168) to 6.3% (*n* = 52/820) (*p* < 0.001); and the overall mortality decreased from 21.4 to 10% (*p* < 0.001). After propensity score matching, 484 correctly triaged and 484 undertriaged patients were identified for subgroup analyses. The in-hospital mortality of undertriaged and correctly triaged patients was 20% (*n* = 99) and 13% (*n* = 61) (*p* = 0.001), respectively.

**Conclusion:**

Undertriage of severely injured patients was associated with significantly increased mortality (20% vs. 13%, *p* = 0.001). Implementation of a trauma center-led education and hotline program coincided with a marked reduction in undertriage rates from 32.7 to 6.3% over the study period. These findings underscore the critical role of regional trauma centers in enhancing prehospital triage through systematic education and real-time medical guidance, particularly in the early phases of trauma system development. Special attention to elderly patients and those with comorbidities is essential for optimizing triage accuracy and reducing preventable trauma deaths.

## Background

Trauma is a leading cause of morbidity and mortality worldwide, underscoring the critical role of efficient emergency medical services (EMS) in prehospital triage and the swift management of severely injured patients [1, 2]. The National Health Insurance Corporation Study conducted in Korea showed that injuries, including trauma, incurred great socioeconomic costs, primarily driven by future income losses due to premature deaths [3]. This is attributed to trauma-related mortality occurring within a relatively younger age demographic compared with other diseases [4]. In the Republic of Korea, trauma is the third leading cause of death and the leading cause of death among individuals younger than 45 years [5]. The preventable trauma death rate in South Korea decreased from 50.4% in 1999 to 39.6% in 2006 [5, 6], prompting the Korean government to promote a government-led trauma centre project since 2010 [6, 7]. During this period, several national-level initiatives were implemented to strengthen the trauma care system. The Korean government designated 17 regional trauma centers between 2012 and 2017, with 15 centers operational by 2021, equipped with Level-I facilities. The Korean Trauma Data Bank (KTDB) was established in 2013 as a nationwide trauma registry to systematically collect clinical data from regional trauma centers. Concurrent improvements in the emergency medical service system included the introduction of multiple dispatch systems for cardiac arrest and severe trauma in 2015, with 79% of cardiac arrest patients and 38% of severe trauma patients receiving multiple dispatches by 2022. These system-wide improvements contributed to a reduction in the national preventable trauma death rate from 30.5% in 2015 to 19.9% in 2017, though regional variations persist [5–7]. 

Correct prehospital triage and direct transportation of severely injured patients to regional trauma centres by EMS providers are pivotal components of a regional trauma system [7–9]. However, accurate triage of patients with severe trauma in the prehospital setting remains challenging [10–13]. Therefore, to improve trauma triage, a regional trauma centre-led training programme for EMS providers, remote medical guidance by experienced trauma staff, and new triage criteria for patients with trauma have been proposed [14–20]. However, EMS providers are insufficiently informed about the Korean Trauma Centre, established in 2014, and paramedics have received no specialised training regarding the triage of patients with severe trauma or the selection of transfer hospitals. Therefore, a programme that provides education and appropriate onsite medical guidance to EMS providers regarding field triage, treatment, and transport is necessary to ensure prompt transportation of patients with severe trauma to trauma centres. As our regional trauma centre has been implementing trauma hotline feedback and case-based education programmes for EMS providers since 2016, we aimed to analyse the effects of these programmes on clinical outcomes.

## Methods

### Aim, design, setting, and population

**Fig. 1:**
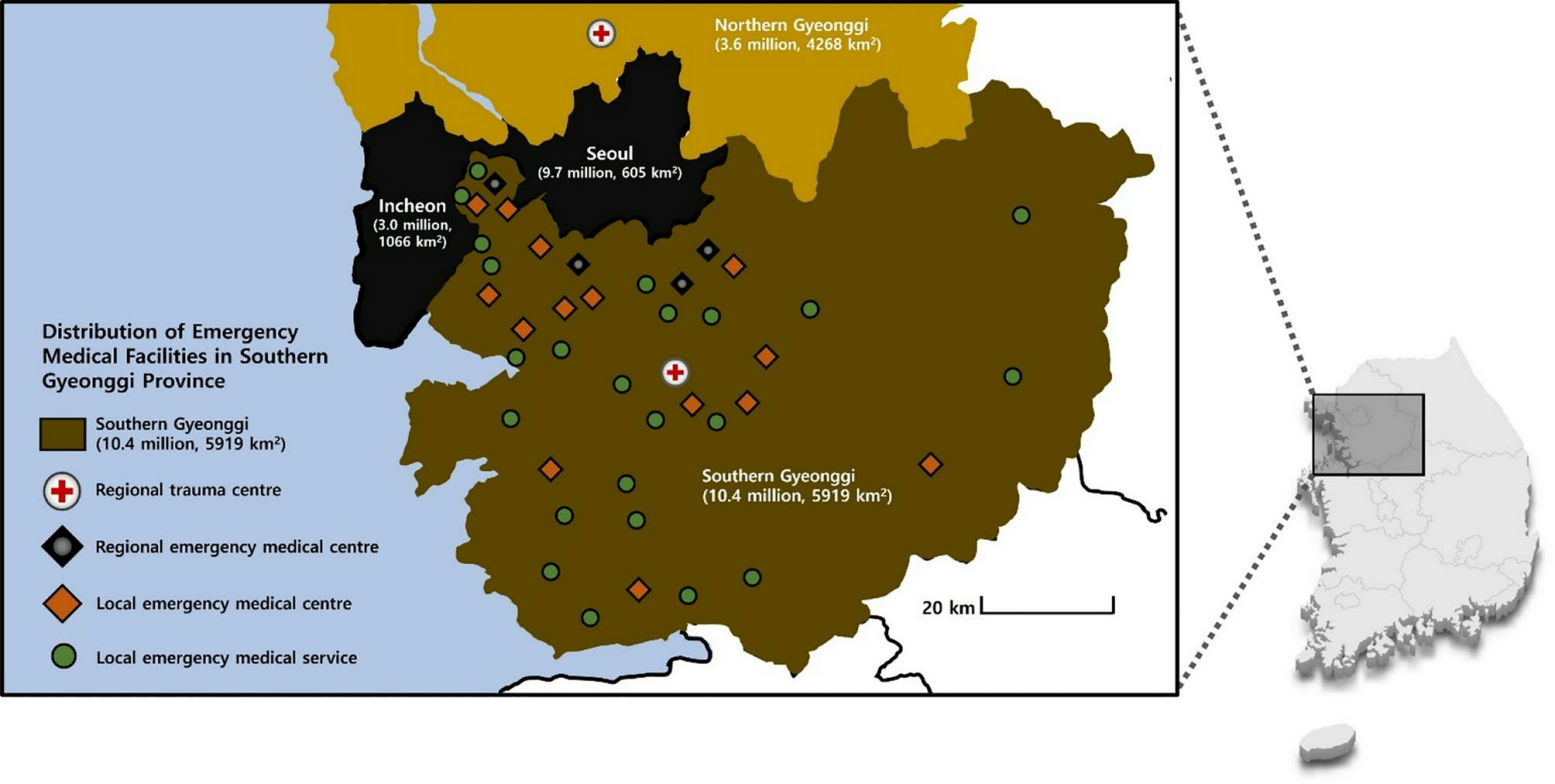
Overview of the southern Gyeonggi province in the Republic of Korea

In retrospective study, we aimed to analyse the effects of regional trauma centre-led feedback and education programmes on clinical outcomes. The Gyeonggi Province has an area of 10,187 km^2^, which is slightly smaller than that of San Diego County, USA (11,720 km^2^; Fig. 1), with a population of 14 million as of 2023, of which 10.38 million (74%) and 3.62 million (26%) live in the southern and northern Gyeonggi Province (5,910 km^2^ and 4,265 km^2^), respectively. The Gyeonggi Province has 57 emergency medical facilities, including five regional emergency medical centres and one trauma centre each in the southern and northern Gyeonggi Provinces. The trauma centre in the southern Gyeonggi Province is in a catchment area, is the only level I trauma centre in the province, and is the final referral institution for patients with severe trauma (Fig. 1). In 2021, 3100 people visited the Trauma Center of Southern Gyeonggi Province, of which 39% (*n* = 1200) had an Injury Severity Score (ISS) > 15. We reviewed the case data of all adult patients with trauma (age ≥ 19 years) with an ISS > 15 who were registered with the Korean Trauma Data Bank and trauma education program record between March 2016 and February 2021 at the Southern Gyeonggi Trauma Center.

### Definition of undertriage

Undertriage can be defined in several ways. Despite several limitations, in general, patients with an ISS > 15 are considered severely injured, and they are deemed to be undertriaged if trauma team activation is not performed [13]. Therefore, in this study, patients with severe injury (ISS > 15) who were not directly transported to the trauma centre were considered undertriaged. Trauma team activation was determined based on documented criteria in patient records, including physiological parameters, anatomical injuries, mechanism of injury, and special considerations.

### EMS provider education and feedback programmes

Since 2016, our regional trauma centre has provided remote medical guidance for EMS providers. In South Korea, EMS providers follow the Field Triage Decision Scheme (https://eymj.org/search.php?where=aview&id=10.3349/ymj.2024.0215&code=0069YMJ&vmode=SM) for identifying patients who require trauma center care. When EMS providers identify patients meeting criteria in Steps 1–4 (physiological, anatomical, mechanism of injury, and special considerations), they are encouraged to contact the regional trauma center directly for medical oversight. Our 24-hour trauma hotline is staffed by trauma surgeons who provide real-time medical guidance.

**Fig. 2:**
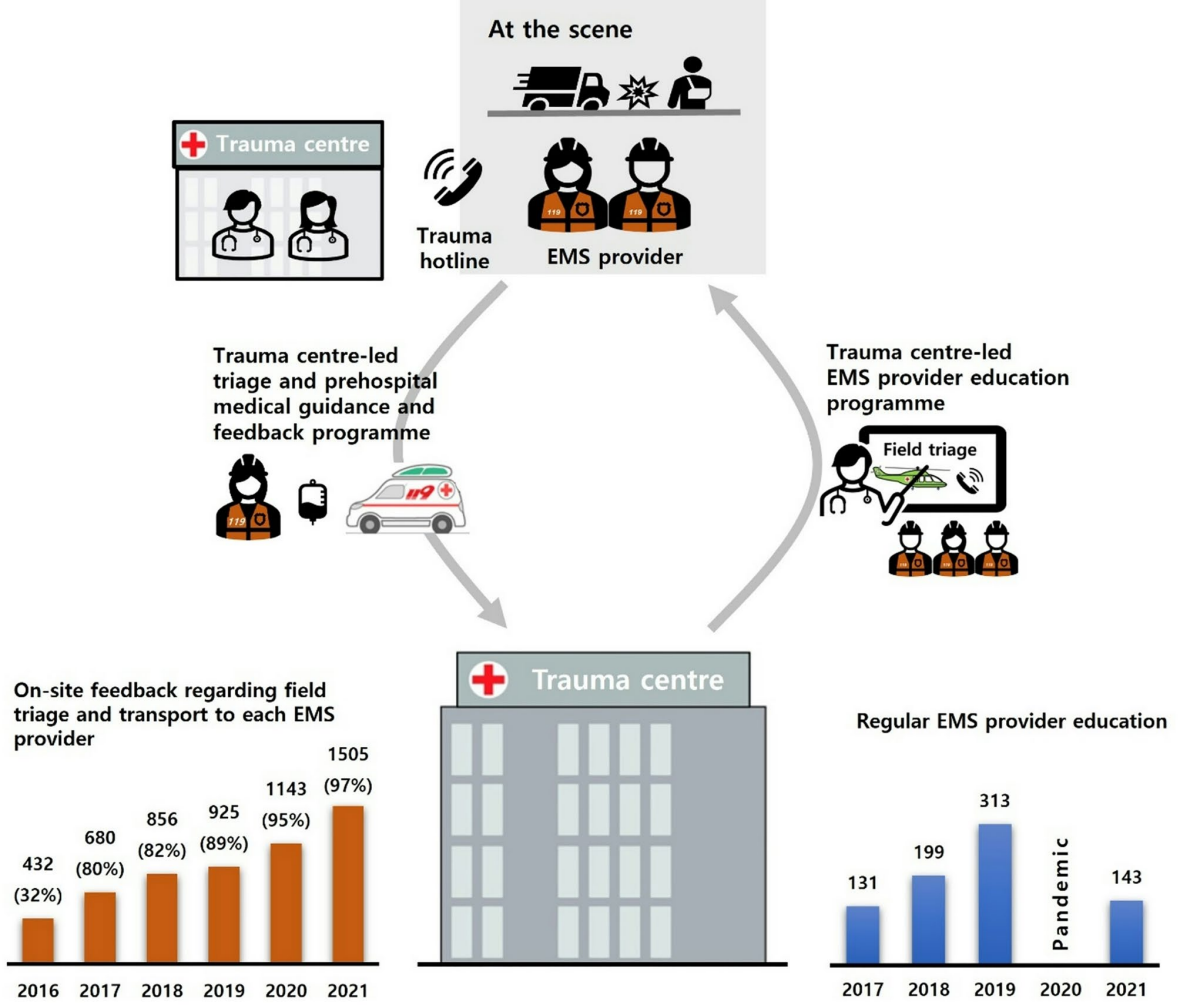
Closed loop model of the regional trauma centre-led performance improvement programme for EMS providers EMS, emergency medical service

Trauma surgeons implementing a 24-hour trauma hotline remotely evaluate the mechanisms of injuries, vital signs, and treatment provided by EMS providers at the scene of the accident. Upon reviewing the provided information, the on-duty trauma surgeon determines whether to deploy the doctor helicopter, by considering the transfer time. The surgeon also guides the approach for airway management and decides on the application of a cervical collar, establishment of intravenous lines, fluid administration, employment of additional haemostasis techniques, and administration of pain management strategies. Immediately after transporting the patient, EMS providers can report the triage, treatment, and transport status to the trauma surgeon and trauma team leader and receive real-time feedback. Through feedback programmes, field triage, first aid, airway management, haemostasis, splinting, and trauma severity assessment are briefly taught, and errors are corrected immediately (Fig. 2). Hotline-based guidance is being provided increasingly frequently every year.

The trauma center implemented a comprehensive education program for EMS providers throughout the study period. The regular education program included annual participation in the International Trauma Conference (5 sessions from 2016 to 2021) and regional EMS provider education programs (25 sessions from 2017 to 2021 with 786 participants). Additional non-regular education programs were conducted based on specific needs, including lectures at the regional fire service academy, Heli-EMS education courses, conferences with the National Fire Agency for regional trauma system establishment, Fire Agency Cooperation Conferences (61 participants), courses for infant and toddler emergency care, field triage and treatment training with hands-on practice (76 participants), standard field skill guideline training, and Trauma Saver Judge competitions (3 sessions from 2019 to 2021). In total, 38 formal education sessions were conducted alongside 5,541 direct admissions and feedback sessions through the trauma bay following prehospital hotline calls. The educational content focused on field triage criteria, initial trauma management, proper use of the trauma hotline, case-based discussions, and hands-on procedural training including airway management, hemorrhage control, and spinal immobilization techniques.

### Covariates and outcomes

Data from the trauma database of the Southern Gyeonggi Province Trauma Center from 2016 to 2021 were analysed. The extracted data included basic demographics, Abbreviated Injury Scale score, ISS, vital signs prehospital and on arrival at the hospital, mechanism of injury, comorbidities, transfusions, transport, admission route, and prehospital medical guidance. Patients with trauma whose dead-on-arrival status and ISS could not be determined and those who visited a hospital outside the Gyeonggi Province were excluded. The primary outcome was the undertriage rate, and the secondary outcomes were in-hospital mortality, hospital length of stay (LOS), intensive care unit LOS, and duration of mechanical ventilation.

### Statistical analyses

Normality was tested using the Kolmogorov–Smirnov test. Continuous variables were compared between the undertriage and correct triage groups using the Mann–Whitney *U* or Student’s *t*-test, and categorical variations were analysed using the chi-squared or Fisher’s exact test. The data are presented as medians and interquartile ranges. Regardless of whether they visited a trauma centre directly, patients with an ISS > 15 who were injured in the Gyeonggi Province were included in an analysis after adjustment for severity and baseline demographics through propensity score matching (PSM) using logistic regression. We performed 1:1 nearest-neighbour matching without replacement with a calliper of 0.2. The propensity score matching value was age, sex, initial systolic blood pressure, injury mechanism and injury severity score (ISS). Absolute standardised mean differences were calculated to evaluate balance, and variables were considered appropriately balanced if the standardised mean difference was < 0.25. For all statistical analyses, SPSS version 23.0 (IBM Corp., Armonk, NY, USA) was used, and for PSM, the MatchIt package for R software version 4.02 (R Foundation for Statistical Computing, Vienna, Austria, 2022) was used. Results were considered statistically significant at *p* < 0.05.

## Results

**Fig. 3:**
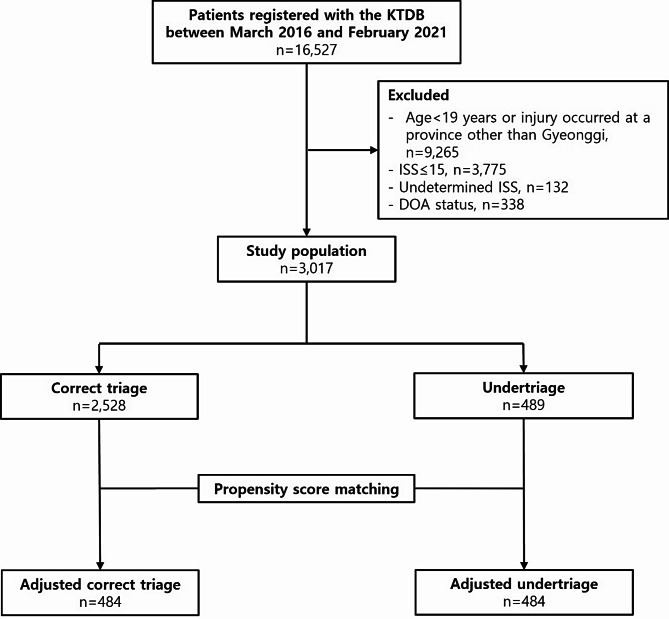
Flowchart depicting the study design KTDB, Korean Trauma Data Bank; ISS, Injury Severity Score; DOA, dead on arrival; PSM, propensity score matching

Between March 2016 and February 2022, 16,527 patients were registered with the Korean Trauma Data Bank in the southern Gyeonggi Province. A total of 7262 patients with trauma aged ≥ 19 years visited the Southern Gyeonggi Trauma Center. After excluding those whose ISS could not be calculated and patients who were dead on arrival, there were 6792 (93% of total) patients, of whom 3017 (41%) patients had an ISS > 15. The 2528 (34% of total) correctly triaged patients visited the trauma centre, whereas 489 (6.7%) undertriaged patients did not visit the centre (Fig. 3). Prehospital medical guidance and feedback, which were conducted 432 times in 2016, increased to 1505 times in 2021. During the study period, 5500 prehospital medical guidance and feedback sessions were conducted (Fig. 2), along with 38 formal education sessions including both regular programs (30 sessions) and non-regular programs (8 sessions) as described in the methods section.

**Fig. 4:**
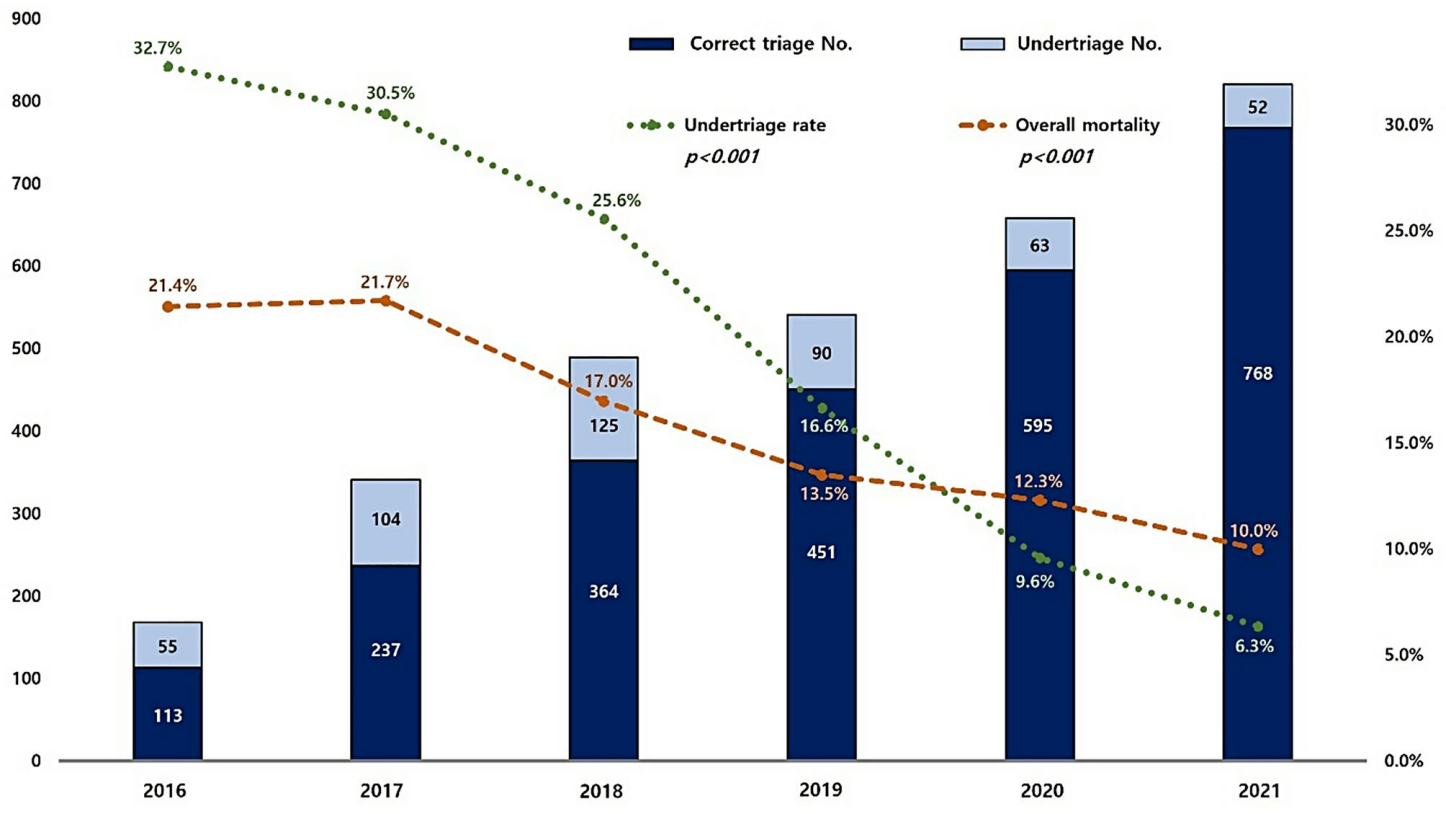
Changes in triage and in-hospital mortality rates by year

Undertriaged patients had a higher mean age (59.4 vs. 50.6 years) and higher comorbidity rates (60.3% vs. 42.7%, *p* < 0.001) than correctly triaged patients (Table 1). The undertriage group comprised more women than men (31.5% vs. 21.0%, *p* < 0.001). The mean Glasgow Coma Scale score was higher in the undertriage group than in the correct triage group (12.8 vs. 11.9, *p* < 0.001). The undertriaged patients had a lower mean ISS (24.8 vs. 26.7, *p* < 0.001) and required a smaller amount of blood product for all types of transfusions for 4–24 h after arrival at the hospital than correctly triaged patients. There were no statistically significant differences between the two groups in terms of systolic blood pressure, pulse rate, or injury mechanisms. From 2016 to 2021, the undertriage rate decreased from 32.7% (*n* = 55/168) to 6.3% (*n* = 52/820) (*p* < 0.001) (Fig. 4).

**Table 1:** General characteristics of the study population and propensity score matched population

	Original population		*p*	Adjusted population		*p*
	Correct_triage	Undertriage		Correct_triage	Undertriage	
	(*n* = 2528)	(*n* = 489)		(*n* = 484)	(*n* = 484)	
**Age**,** years**	52 [37–63]	60 [49–74]	< 0.001	60 [47–74]	60 [48–74]	*0.817*
19–64	1993 (78.8%)	297 (60.7%)	< 0.001	298 (61.6%)	297 (61.4%)	*1.000*
≥ 64	535 (21.2%)	192 (39.3%)	< 0.001	186 (38.4%)	187 (38.6%)	*1.000*
**Sex**			< 0.001			*0.449*
Male	1997 (79.0%)	335 (68.5%)		323 (66.7%)	335 (69.2%)	
Female	531 (21.0%)	154 (31.5%)		161 (33.3%)	149 (30.8%)	
**ISS**	24 [19–33]	25 [17–26]	< 0.001	22 [18–29]	25 [17–26]	*0.132*
16–30	1860 (73.6%)	412 (84.3%)	< 0.001	377 (77.9%)	407 (84.1%)	*0.018*
≥ 30	668 (26.4%)	77 (15.7%)	< 0.001	107 (22.1%)	77 (15.9%)	*0.018*
**Injury mechanism**			0.209			*1.000*
Blunt	2434 (96.3%)	477 (97.5%)		473 (97.7%)	472 (97.5%)	
Traffic accident	1439 (56.9%)	168 (34.4%)		290 (59.9%)	168 (34.7%)	
Fall	122 (30.3%)	99 (20.2%)		128 (26.4%)	97 (20.0%)	
Ground fall	40 (1.6%)	122 (24.9%)		15 (3.1%)	119 (24.6%)	
Machine	32 (1.3%)	2 (0.4%)		5 (1.0%)	2 (0.4%)	
Struck	112 (4.4%)	23 (4.7%)		24 (5.0%)	23 (4.8%)	
Others	45 (1.8%)	63 (12.9%)		11 (2.3%)	63 (13.0%)	
Penetrating	94 (3.7%)	12 (2.5%)		11 (2.3%)	12 (2.5%)	
**Time to trauma center**,** min**	48 [35–63]	109 [60–221]	< 0.001	49 [34–62]	108 [40–118]	*< 0.001*
**Hospital value on arrival**						
AVPU scale (*n* = 3017)			< 0.001			*< 0.001*
- Alert	1127 (44.6%)	239 (48.9%)		208 (43.0%)	235 (48.6%)	
- Verbal response	615 (24.3%)	76 (15.5%)		134 (27.7%)	76 (15.7%)	
- Painful response	507 (20.1%)	99 (20.2%)		102 (21.1%)	99 (20.5%)	
- Unresponsive	279 (11.0%)	75 (15.3%)		40 (8.3%)	74 (15.3%)	
SBP (mmHg, *n* = 2688)	134 [117–153]	135 [115–158]	0.656	137 [120–155]	135 [115–158]	*0.539*
PR (min^− 1^, *n* = 2878)	88 [75–103]	86 [76–100]	0.232	86 [72–100]	86 [76–99]	*0.441*
GCS (*n* = 2773)	14 [9–15]	15 [11–15]	< 0.001	14 [10–15]	15 [11–15]	*< 0.001*
**Transfusion (unit)**						
PRBC_24h	0 [0–4]	0 [0–2]	< 0.001	0 [0–4]	0 [0–2]	*0.005*
FFP_24h	0 [0–4]	0 [0–2]	0.016	0 [0–3]	0 [0–2]	*0.25*
Platlet_24h	0 [0–0]	0 [0–0]	0.377	0 [0–0]	0 [0–0]	*0.077*

*ISS*, Injury Severity Score; *SBP*, systolic blood pressure; *PR*, pulse rate; *GCS*, Glasgow Coma Scale; *PRBC*, packed red blood cells; *FFP*, fresh frozen plasma

### Primary and secondary outcomes

**Fig. 5:**
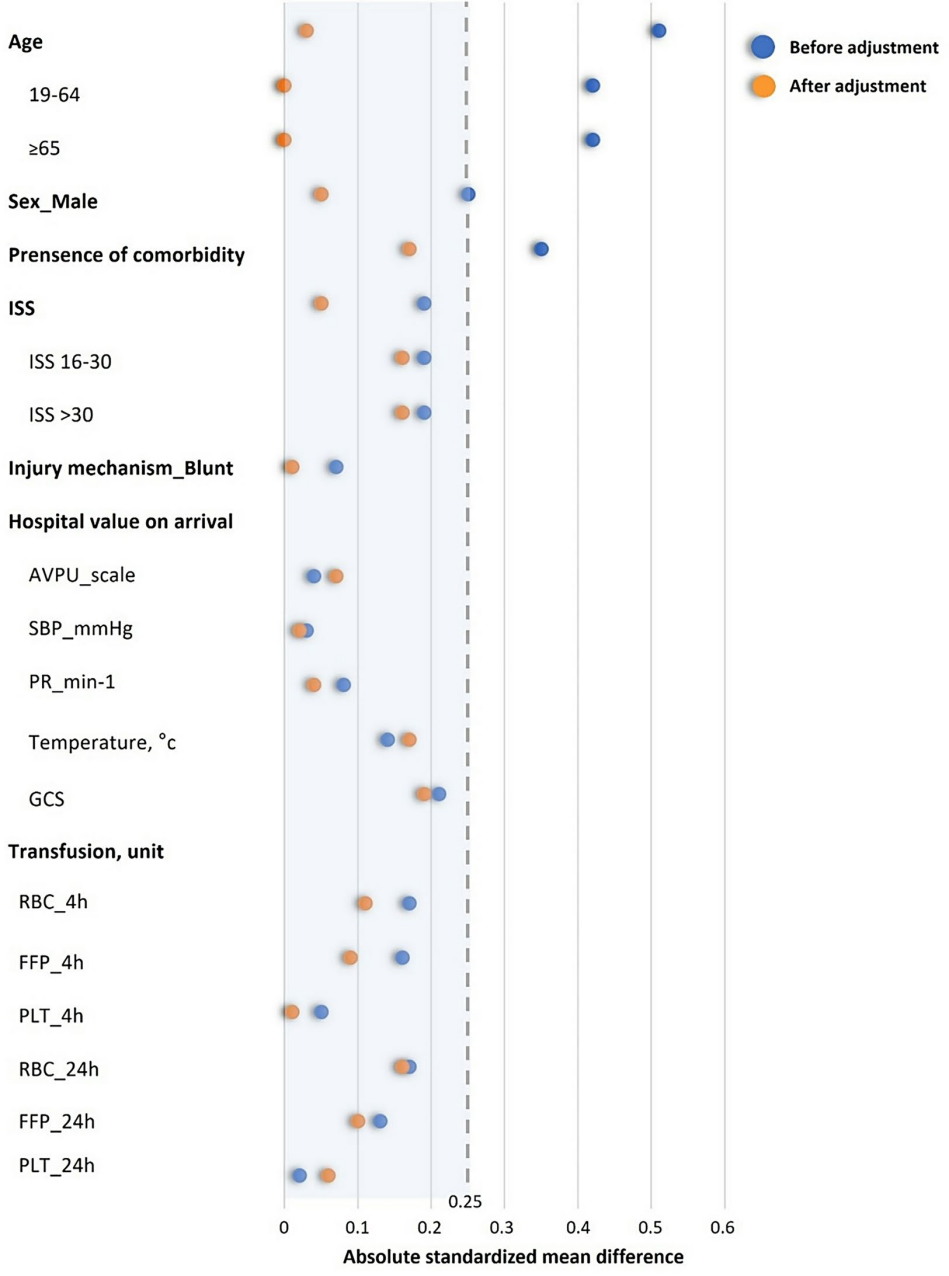
Standardised mean differences before and after adjustment of covariates GCS, Glasgow Coma Scale; RBC, red blood cells; FFP, fresh frozen plasma; PLT, platelet

To analyse the primary outcome (undertriage), PSM was performed to adjust for demographic variables and trauma severity. Standardised mean differences were calculated to evaluate balance, and all variables were appropriately balanced (Fig. 5). After propensity score matching, specific comorbidities remained significantly associated with undertriage (Table 2). The in-hospital mortality rate in the adjusted cohort was 20% (*n* = 99) for undertriage and 13% (*n* = 61) for correct triage (*p* = 0.001) (Table 3). The median (interquartile range) hospital LOS was 17 (8–37 days) and 19 (12–36 days) (*p* = 0.024) and the median intensive care unit day ratio was 0.4 (0.2–1.0 days) and 0.3 (0.2–0.6 days) (*p* = 0.059) for undertriage and correct triage, respectively. The duration of mechanical ventilation did not show a statistically significant difference between the groups (Table 3). The median time until death of the patients was 3.2 days, and the correct triage group excluding deaths had lower hospital LOS (20 [14–39 vs. 23 [12–40], *p* = 0.756) and ICU LOS (5 [3–11] vs. 6 [3–17], *p* = 0.466), but statistical significance could not be confirmed (Table 3).

**Table 2:** Comorbidities of the study population and propensity score matched population

	Original population			Adjusted population		*p*
	Correct triage	Undertriage	*p*	Correct triage	Undertriage	
	(*n* = 2528)	(*n* = 489)		(*n* = 484)	(*n* = 484)	
Myocardial Infarction	14 (0.6%)	6 (1.2%)	*0.169*	3 (0.6%)	6 (1.2%)	*0.503*
Congestive Heart Failure	0 (0.0%)	1 (0.2%)	*0.359*	0 (0.0%)	1 (0.2%)	*1.000*
Peripheral Vascular Disease	2 (0.1%)	2 (0.4%)	*0.248*	0 (0.0%)	2 (0.4%)	*0.479*
Cerebrovascular Disease	71 (2.8%)	49 (10.0%)	***< 0.001***	16 (3.3%)	48 (9.9%)	***< 0.001***
Dementia	24 (0.9%)	19 (3.7%)	***< 0.001***	10 (2.1%)	19 (3.9%)	*0.131*
COPD	2 (0.1%)	2 (0.4%)	*0.248*	1 (0.2%)	2 (0.4%)	*1.000*
Connective Tissue Disease	4 (0.2%)	0 (0.0%)	*0.840*	0 (0.0%)	0 (0.0%)	*1.000*
Peptic Ulcer Disease	4 (0.2%)	0 (0.0%)	*0.840*	1 (0.2%)	0 (0.0%)	*1.000*
Diabetes Mellitus	321 (12.7%)	93 (19.0%)	***< 0.001***	83 (17.1%)	91 (18.8%)	*0.558*
Chronic Kidney Disease	16 (0.6%)	12 (2.5%)	***< 0.001***	6 (1.2%)	12 (2.5%)	*0.234*
Hemiplegia	1 (0.0%)	2 (0.4%)	*0.112*	1 (0.2%)	2 (0.4%)	*1.000*
Leukemia	1 (0.0%)	1 (0.2%)	*0.736*	0 (0.0%)	1 (0.2%)	*1.000*
Malignant Lymphoma	1 (0.0%)	1 (0.2%)	*0.736*	0 (0.0%)	1 (0.2%)	*1.000*
Solid Tumor	47 (1.9%)	28 (5.7%)	***< 0.001***	11 (2.3%)	27 (5.6%)	***0.013***
Liver Disease	57 (2.3%)	10 (2.0%)	*0.904*	10 (2.1%)	10 (2.1%)	*1.000*
AIDS	0 (0.0%)	1 (0.2%)	*0.359*	0 (0.0%)	1 (0.2%)	*1.000*
Bleeding Disorder	1 (0.0%)	1 (0.2%)	*0.736*	0 (0.0%)	1 (0.2%)	*1.000*
Angina	17 (0.7%)	10 (2.0%)	***0.007***	5 (1.0%)	10 (2.1%)	*0.298*
Hypertension Requiring Medication	556 (22.0%)	167 (34.2%)	***< 0.001***	142 (29.3%)	166 (34.3%)	*0.112*
Steroid Use Oral	7 (0.3%)	2 (0.4%)	*0.970*	2 (0.4%)	2 (0.4%)	*1.000*
Major Psychiatric Disorder	80 (3.2%)	6 (1.2%)	***0.027***	18 (3.7%)	6 (1.2%)	***0.023***

*COPD*, Chronic Obstructive Pulmonary Disease; *AIDS*, Acquired immune deficiency syndrome

**Table 3:** In-hospital outcomes of correct triage vs. undertriage

	Original population			Post PSM population		
	Correct_triage	Undertriage	*p*	Correct_triage	Undertriage	*p*
	(*n* = 2528)	(*n* = 489)		(*n* = 484)	(*n* = 484)	
Hospital LOS, day	18 [11–35]	17 [8–36]	*0.023*	19 [12–36]	17 [8–37]	*0.024*
Except mortality	21 [13–37]	23 [12–40]	*0.991*	20 [14–39]	23 [12–40]	*0.756*
ICU_LOS, day	5 [3–12]	5 [2–15]	*0.856*	5 [3–11]	5 [2–16]	*0.811*
Except mortality	5 [3–12]	6 [3–17]	*0.378*	5 [3–11]	6 [3–17]	*0.466*
MV_duration, day	1 [0–6]	1 [0–8]	*0.459*	1 [0–5]	1 [0–8]	*0.190*
Except mortality	0 [0–5]	0 [0–8]	*0.420*	0 [0–4]	0 [0–8]	*0.109*
Inhospital death (%)	329 (13%)	100 (20%)	*0.000*	61 (13%)	99 (20%)	***0.001***
Time to death, day	3.0 [0.4–10.6]	3.3 [0.2–10.8]	0.521	3.4 [0.8–8.7]	3.4 [0.2–10.8]	*0.109*

*PSM*, propensity score matching; *LOS*, length of stay; *MV*, mechanical ventilator

## Discussion

This study showed that undertriage was associated with increased in-hospital mortality and hospital LOS. Moreover, the prehospital medical guidance significantly increased and the undertriage rate significantly decreased from 2016 to 2021.

Appropriate treatment at the right time and place is important to improve the survival rate of patients with severe trauma [21]. The American College of Surgeons Committee on Trauma recommends an undertriage rate target of 5% or less in regional trauma systems [21, 22]. Although differences by region may exist and the system might not be the same in other countries, the EMS provider performs the initial trauma triage in South Korea [4, 23]. Therefore, the undertriage rate is determined by examining the hospital selection based on the initial triage by the EMS provider [23]. Even though triaging patients with trauma is difficult, areas with well-developed trauma systems can have high undertriage rates [11, 13]. General physicians, including emergency physicians, report that triage accuracy is low if the EMS provider has insufficient experience with patients with severe trauma [12]. Therefore, to reduce the undertriage of patients with severe trauma, various educational programmes, medical guidance programmes, mobile application developments, and revisions of the field triage scheme have been attempted for EMS providers, nurses, and physicians who perform field triage [14–20, 24–26]. In the southern Gyeonggi Province a government-led designated trauma centre was established in 2016, and a regional trauma centre-led programme that educates and provides medical guidance to EMS providers was subsequently initiated. Trauma surgeons man a 24-hour trauma hotline, remotely evaluate the mechanisms of injuries, vital signs, and treatment provided by EMS providers at the scene of the accident, and provide medical advice on field triage, treatment, and transportation. Immediately after transporting the patient, EMS providers can report triage, treatment, and transport to the trauma surgeon and trauma team leader and receive immediate feedback. Prehospital medical guidance and feedback were conducted 432 times in 2016, which increased to 1505 times in 2021; they were conducted a total of 5541 times during the study period. The institutional undertriage rate decreased from 32.7% in 2016 to 6.3% in 2021.

According to the American College of Surgeons Committee on Trauma, the criteria for distinguishing between level 1 and level 2 trauma centres are resources, trauma volume, and educational commitments [27]. Level I trauma centres maintain in-house availability of resuscitation, operative procedures, and critical care, in which attending trauma surgeons participate 24 h a day, hospitalise more than 240 patients with trauma with ISSs ≥ 16 annually, and perform trauma education, training, and research. In implementing the regional trauma system, it is important to improve the ability of EMS providers to perform prehospital triage of patients with trauma [23, 28]. The Korean Fire Agency partially supports the triage of patients with trauma and medical guidance by general or emergency physicians; however, the accuracy of triage by medical staff who do not have much experience with patients with severe trauma may not be high [12]. Chiu et al. reported that field triage training for EMS providers can improve triage accuracy; the authors have been operating a trauma education programme for local EMS providers since 2017. We conducted various educational programmes, in which we discussed cases of patients with severe trauma triaged and treated by EMS providers. In all training sessions, the trauma hotline was introduced, and information was provided that enabled the EMS providers to communicate with a trauma surgeon 24 h a day about field triage, medical guidance, and transport for patients with suspected severe trauma.

Performance improvement programmes based on the closed-loop principle have been widely adopted to improve trauma management quality [29–33]. Huh et al. reported improvements in in-hospital outcomes, such as the preventable trauma death rate after implementing a performance improvement and patient safety programme based on the closed-loop principle at a new regional trauma centre [29]. Hietbrink et al. studied the Netherlands’ Trauma System and reported that the odds ratio for mortality in level I trauma centres decreased to 0.54 as the number of correct triage procedures increased [34]. To increase the correct triage rate, the EMS providers’ education and feedback programme implemented by the regional trauma centre in this study enables communication between regional trauma centres and EMS providers, according to the closed-loop principle. The undertriage rate decreased from 32.7% in 2016 to 6.3% in 2021, and the overall mortality rate decreased from 21.4 to 10.0%. The in-hospital mortality rate of the correct triage group was 0.56 times that of the undertriage group. The median time to death was 3.2 days, which may lead to a selection bias in which the LOS of the undertriage group with many deaths is reduced. Therefore, when the LOS was analyzed after excluding the deceased patients, the length of hospital stay and intensive care unit stay in the correct triage group were confirmed to be shorter, but no statistical difference could be confirmed.

Our study, which showed a higher tendency for undertriage in female patients, is consistent with the results of studies by Rubenson [35], Holst [36], and Xiang [11]. Rubenson [35] analyzed that the reason for the higher tendency for undertriage in women was the higher proportion of low-energy injury mechanisms. In our study, low-energy trauma such as ground fall was the third most common injury mechanism in women (11%, *n* = 73), whereas it was the fifth most common injury mechanism in men (2%, *n* = 35). In other words, the frequency of severely injured (ISS > 15) due to low-energy trauma in women was more than five times higher than in men.

Our propensity score matched analysis revealed important insights regarding elderly patients and comorbidities as risk factors for undertriage. Advanced age remains a significant predictor, with the undertriage group showing higher mean age (60 vs. 52 years, *p* < 0.001). This aligns with extensive literature demonstrating systematic undertriage of older trauma patients due to age-related physiological changes that mask injury severity. Benjamin et al. [13] showed that patients ≥ 60 years with comorbidities like CHF or CVA have significantly increased risk of early mortality despite apparent physiological stability. Specific comorbidities remained significantly associated with undertriage even after propensity score matching (Table 2): - Cerebrovascular disease (9.9% vs. 3.3%, *p* < 0.001): Patients with prior stroke may have altered mental status baselines, making GCS assessment challenging and leading to underestimation of acute injury severity - Solid tumors (5.6% vs. 2.3%, *p* = 0.013): These patients may preferentially seek care at their familiar oncology centers rather than trauma centers, contributing to undertriage - Major psychiatric disorders (1.2% vs. 3.7%, *p* = 0.023): Interestingly, psychiatric patients were less likely to be undertriaged, possibly due to mandatory transport protocols in our region for psychiatric emergencies The persistence of these associations after matching suggests that comorbidity-specific factors, beyond simple age or injury severity, influence triage decisions. Previous studies have shown that comorbidities and medications affect both injury patterns and physiological responses, with current triage tools showing poor sensitivity for identifying severe injuries in patients with multiple comorbidities [11, 37, 38]. This supports the need for EMS education to specifically address the assessment challenges posed by comorbid conditions and emphasizes the importance of considering patient complexity in triage decisions.

Our study had several limitations. First, our study focused exclusively on undertriage of severely injured patients and did not examine overtriage patterns. While resource utilization is important, our priority was addressing the patient safety concerns associated with undertriage in our developing trauma system. Second, we could not capture complete data on secondary transfers, as our registry primarily includes patients who arrived at our center. Among undertriaged patients, we could only track outcomes for those eventually transferred to our facility (58.7%), which may introduce selection bias. Third, this study may have a selection bias owing to its single-institution retrospective design. Therefore, a multicentre study is required to verify these results. However, since the establishment of the regional trauma centre, improvements in the preventable trauma death rate and the development of the regional trauma system have been quantitatively confirmed [39]. Therefore, the authors believe that this study serves as evidence that the regional trauma centre-led performance improvement programme contributed to the improvement of the undertriage rate and development of the regional trauma system. Lastly, the method of retrospectively evaluating triage accuracy based on the Abbreviated Injury Scale and ISS at regional trauma centres may underestimate the actual severity because it does not reflect the physiological, anatomical, or mechanism of injury criteria of the field triage scheme proposed by the Centers for Disease Control and others [10, 24, 40]. However, for the convenience of trauma research and quality management of trauma treatment, the method of evaluating the accuracy of triage based on the ISS is widely adopted [10, 26, 41].

## Conclusions

Undertriage of severely injured patients was associated with significantly higher mortality, emphasizing its critical impact on trauma outcomes. Our study demonstrated that implementation of a comprehensive trauma center-led education and hotline program coincided with a substantial reduction in undertriage rates from 32.7 to 6.3%, approaching the recommended target of less than 5%. These findings highlight the vital role of regional trauma centers in improving prehospital triage accuracy through systematic education, real-time medical guidance, and continuous feedback. Special attention should be directed toward elderly patients, women with low-energy trauma mechanisms, and patients with specific comorbidities who remain at higher risk for undertriage. Future prospective studies are needed to establish causality and develop comorbidity-adjusted triage protocols to further reduce preventable trauma mortality.

## Data Availability

No datasets were generated or analysed during the current study.

## Abbreviations

DOA: Dead on arrival
EMS: Emergency medical service
FFP: Fresh frozen plasma
GCS: Glasgow Coma Scale
ICU: Intensive care unit
ISS: Injury Severity Score
KTDB: Korean Trauma Data Bank
LOS: Length of stay
MV: Mechanical ventilation
PLT: Platelet
PR: Pulse rate
PRBC: Packed red blood cells
PSM: Propensity score matching
RBC: Red blood cells
SBP: Systolic blood pressure
