# Correction: Right patient to the right place: the impact of a 6-year regional trauma centre-led prehospital education programme on EMS triage and patient outcomes

**DOI:** 10.1186/s12873-025-01367-w

**Published:** 2025-09-29

**Authors:** Donghwan Choi, Yo Huh, Byung Hee Kang, Sora Kim, Seoyoung Song, Kyoungwon Jung, Hohyung Jung

**Affiliations:** 1 Division of Trauma Surgery, Department of Surgery, Ajou University School of Medicine, 164 Worldcup-ro, Yeongtong-gu, Suwon-si, Gyeonggi-do 16499 Republic of Korea; 2 Regional Trauma Center of Southern Gyeonggi Province, Ajou University School of Medicine, Suwon, Republic of Korea

**Correction: BMC Emergency Medicine (2025) 25:159**

10.1186/s12873-025-01321-w

The original article has been corrected to correct a typo in author, Hohyung Jung’s name.

